# An Exploration of the Impact of Anticentromere Antibody on Early-Stage Embryo

**DOI:** 10.1155/2017/4809294

**Published:** 2017-10-04

**Authors:** Ying Ying, Xi Guo, Yiping Zhong, Canquan Zhou

**Affiliations:** ^1^Reproductive Medicine Center, The Third Affiliated Hospital of Guangzhou Medical University, Guangzhou, China; ^2^Reproductive Medicine Center, The First Affiliated Hospital of Sun Yat-sen University, Guangzhou, China

## Abstract

**Background:**

Previously, we found women with positive anticentromere antibody showed impaired potential of oocyte maturation and embryo cleavage; the possible mechanism behind this phenomenon was still unknown.

**Objective:**

Thus, the present study aimed to preliminarily explore whether ACA could penetrate into the living embryos and impair their developmental potential via *in vitro* coculture with mouse embryos.

**Methods:**

Mouse embryos were collected and used for *in vitro* culture with polyclonal anticentromere protein A (CENP-A) antibody; then, immunofluorescence assay was performed to determine the penetration of antibody into embryos, and embryo development potential was observed.

**Results:**

All embryos cultured with anti-CENP-A antibody exhibited immunofluorescence on the nucleus, while none of the embryos from the control groups showed immunofluorescence. Additionally, embryos cultured with anti-CENP-A antibody experienced significant growth impairment compared with controls.

**Conclusion:**

Mouse embryos may be a direct target for ACA *in vitro* prior to implantation. However, the precise mechanism needs further clarification.

## 1. Introduction

A recent study revealed disorders of oocyte maturation and early embryonic development in women with positive anticentromere antibody (ACA) in their peripheral blood [1]. More recently, we found that women positive for ACA had a significantly lower percentage of mature oocytes and embryo cleavage rate compared with women negative for ACA [2], further revealing the potential impact of ACA on female fertility. ACA is known to be one of the members of ANAs. It was first discovered in 1980 as a specific antibody against centromere in serum of patients with calcinosis, Raynaud's phenomenon, esophageal dysmotility, sclerodactyly, and telangiectasia (CREST) syndrome [3, 4]. Now, ACA has been recognized as an effective auxiliary diagnostic marker for systemic sclerosis (SSc). As reported, female patients with SSc are susceptible to have several different adverse pregnancy outcomes, including increased spontaneous abortion rate, premature birth, small babies, and infertility [5, 6]. Additionally, the infertility prevalence in patients with SSc is high, and the success rate for infertility treatment is relatively low, which needs further investigation [7].

As early as the 1990s, researchers attempted to microinject ACA into mouse eggs, which led to disorders of chromosomal movement and segregation [8]. It is known that kinetochore is the attachment site of spindle microtubules in the centromeric region of a chromosome [9, 10]. Also, it is the dynamic structure for mitosis, meiosis, and other important activities of cells [11–15]. Therefore, it would be reasonable to infer that ACA might interfere with meiosis or mitosis in living cells.

Centromere is a DNA-protein complex, and its assembly is coregulated by centromeric chromatins and their associated protein complex [16, 17]. Centromere protein A (CENP-A) is one of the constitutive centromere proteins with relatively clear biological functions that has been mostly studied; its important role in assembly and functional implementation of centromere has been repeatedly verified [18, 19]. Furthermore, similar to CENP-B, CENP-A is considered to be a major target antigen of ACA [20–23].

It was speculated that ACA might be one of the ANAs most closely associated with abnormal oocyte maturation and embryo cleavage. Therefore, the aim of the present study was to explore the potential impact of ACA on early-stage embryos via *in vitro* coculture with mouse embryos.

## 2. Materials and Methods

### 2.1. Mouse Embryos

Superovulation was induced in outbred ICR mice by stimulating with pregnant mare's serum gonadotrophin (10 IU intraperitoneally (i.p.)) and human chorionic gonadotrophin (10 IU i.p. after 48 h) and mated with ICR males. The female mice were killed 24 h after mating. Early-stage embryos were collected by sharp dissection of the fallopian tubes and used in the experiments. The Ethics Committee of the First Affiliated Hospital of Sun Yat-Sen University approved this study.

### 2.2. *In Vitro* Embryo Culture

The embryos were cultured in the Quinn's serial medium (Sage, USA). For the antibody group, rabbit polyclonal antibody to mouse CENP-A (bovine serum albumin and azide free, customized products from Abcam, United Kingdom) was added to the medium. The antibody concentration in the medium was 35 *μ*g/mL (modified based on the literature [24]). For the phosphate-buffered saline (PBS) group (served as controls), the PBS solution (PBS tablet, Millipore, Merck, Germany) with the same volume as the antibody solution was added to the medium. The blank control group comprised the medium without any additives.

### 2.3. Immunofluorescence Assay

On the second and third days of culture, three to five embryos were picked from each dish of the three groups for the immunofluorescence assay, to detect whether the signals of anti-CENP-A antibody were present in the embryos after coculture. The procedures for the immunofluorescence assay were as follows: The embryos were fixed in 4% polyoxymethylene and then permeated with 0.5% Triton X-100 (Sigma, USA), followed by sealing in 5% normal donkey serum (Jackson Immunoresearch, USA). After that, the embryos were incubated for 1 h with 488-labeled donkey antirabbit IgG (Invitrogen, UK, 1 : 1000 dilution), rinsed, incubated with 1 *μ*g /mL DAPI (4′,6-diamidino-2-phenylindole, dihydrochloride, Cell Signaling Technology, USA) for 15 min, again rinsed, and fixed in a dish for subsequent microscopic observation. In order to rule out false positive of the experimental group, embryos from antibody group were incubated with PBS instead of 488-labeled donkey antirabbit IgG (antibody group for control).

### 2.4. Development of Cocultured Embryos (Embryotoxicity Assay)

The collection and culturing of the embryos for this embryotoxicity assay were the same as described earlier except that the embryos remained in the dishes for the entire 3-day period. The embryos in each group were examined to determine their stage of development on the third and fifth days (the first day referred to the day of oocyte collection). The following developmental stages were recorded: 6- to 8-cell stages on the third day, and blastocyst, morula, 2- to 8-cell, and atretic stages on the fifth day.

### 2.5. Statistical Analysis

Statistical analysis was performed using SPSS 13 (SPSS, IL, USA). A chi-square test and partition of chi-square tests were used to compare qualitative data. A *P* value less than 0.05 was considered statistically significant by chi-square test among the three groups, and a *P* value less than 0.0167 was used to indicate statistical significance in the partition of chi-square tests between groups.

## 3. Results

### 3.1. Immunofluorescence

**Figure 1:**
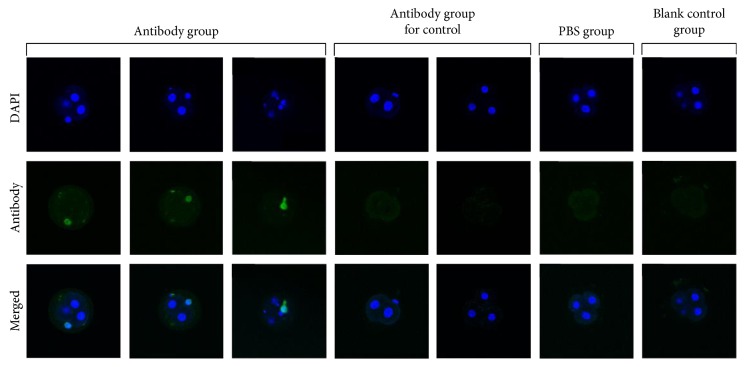
Immunofluorescence views of embryos cultured with anti-CENP-A antibody (original magnification ×400). Antibody fluorescence was present on the nuclei of blastomeres in embryos from the antibody group, while no significant antibody fluorescence was observed in the embryos from groups of PBS, blank control, and antibody group for control.

All embryos cultured with anti-CENP-A antibody exhibited strong immunofluorescence in their nuclei, while none of the embryos from the PBS and blank control groups, as well as the antibody group for control, showed immunofluorescence (Figure 1).

### 3.2. Embryotoxicity Assay

Compared with the PBS and blank control groups, the percentages of 6- to 8-cell stage embryos on the third day, as well as blastula and morula stage embryos on the fifth day, were significantly lower in the antibody group. The developmental potentials of embryos were comparable between the PBS and blank control groups (Table 1).

## 4. Discussion

In 1999, researchers found that all mouse embryos cultured with purified antinuclear IgG exhibited strong immunofluorescence on the embryonic cells and experienced significant growth impairment or death compared with those cultured with control immunoglobulins [25]. This indicated that ANAs could bind directly to embryos *in vitro*. However, the precise epitopes were not known because no nuclear antigens or phospholipids were found in the zona. In mouse 2-cell stage embryos, a set of nucleoproteins is transiently synthesized and changes in embryonic chromatin composition, suggesting that early embryos may possess epitopes for ANA [26]. Furthermore, the binding is relatively specific, as antithyroid antibody and antibodies from healthy individuals show no evidence of binding to embryos. Microinjection of serum containing ACA into mouse oocytes could hinder chromosome congression and cause meiotic arrest in interphase or mitotic arrest in prometaphase [8].

In the present study, all embryos cultured with polyclonal anti-CENP-A antibody showed strong immunofluorescence of antibody against nuclear components (which were speculated to be anti-CENP-A antibody) and experienced apparent embryonic growth impairment, indicating that mouse embryos may be a direct target for some ACAs *in vitro* prior to implantation. In addition, for the majority of cocultured embryos, always only one or some of the blastomeres showed fluorescence. Perhaps, the density of structures in and around the centromere prevents anti-CENP-A antibody accessibility, or the blastomere with detectable fluorescence was inclined to apoptosis and displayed relatively loose structures that enabled anti-CENP-A antibody accessibility. However, the precise mechanism needs further clarification.

Although no definite concept exists for antibody entering the living cells, and the mechanism involved is unknown yet, these studies provided evidence for antibodies entering the living cells. For example, the anti-ribonucleoprotein-IgG could selectively enter the T-lymphocytes, while relatively less Fc receptors were present on the surface of T-lymphocytes. It suggested that some other mechanisms relevant to non-Fc regulation might be involved, which might be associated with antigen-like structures on the lymphocyte surface, implying that the ribonucleoprotein antibodies interacted with the ribonucleoprotein antigens on the cell surface to regulate their access into the living cells [27].

This study also found that the embryonic developmental potential was significantly impaired after culture with anti-CENP-A antibody, exhibiting significantly lower percentages of 6- to 8-cell stage embryos on the third day and blastula stage embryos on the fifth day. In fact, a previous study found that mouse embryos cultured with purified IgG from ANA-positive serum showed significantly impaired embryonic development potential [25]. ANA could penetrate into subcellular structures containing corresponding antigens, where it could identify and bind to epitopes in the important functional regions. These autoantibodies could inhibit significantly the function of antigens both in vivo and *in vitro* [28].

In conclusion, embryos cultured with anti-CENP-A antibody experienced significant growth impairment or death. Thus, embryos could be a direct target for anti-CENP-A antibody.

## Figures and Tables

**Table 1:** Embryotoxicity assay.

Parameters (%)	Antibody group	PBS group	Blank control group	*P* value
6- to 8-cell stages on the third day	24.8% (114/459)	48.4% (183/378)	51.0% (192/376)	<0.01^a,b^
Blastula stage on the fifth day	26.8% (123/459)	57.1% (216/378)	64.9% (244/376)	<0.01^a,b^
Morula stage on the fifth day	12.4% (57/459)	24.6% (93/378)	20.2% (76/376)	<0.01^a,b^

The first day referred to the day of embryo collection. *P* < 0.05 was considered statistically significant among the three groups. ^a^*P* < 0.0167 versus antibody and PBS groups. ^b^*P* < 0.0167 versus antibody and blank control groups.
